# Adventitious agent contamination risk mitigation: engineering MMV virus resistance into CHO cells

**DOI:** 10.1186/1753-6561-9-S9-O2

**Published:** 2015-12-14

**Authors:** Joaquina X Mascarenhas, Lisa Burger, Ademola Kassim, Trissa Borgschulte, Delia Lyons, Henry George, Nan Lin, Audrey Chang, David Onions, David Pintel, Kevin Kayser

**Affiliations:** 1 Cell Sciences and Development, SAFC Sigma Aldrich, Saint Louis, Missouri, 63117, USA; 2 School of Medicine, University of Missouri, Columbia, Missouri, 65211, USA; 3 Bioreliance, SAFC Sigma Aldrich, Rockville, Maryland, 20850, USA

## Background

The introduction of animal origin free (AOF) media has significantly reduced the incidence of adventitious virus contamination in biological production systems. Nevertheless, contamination by the parvovirus Mouse Minute Virus (MMV) remains a continuing challenge. Although infrequent, infection of a fermenter can be catastrophic for a manufacturer, and can also have a potential impact on drug supply, patient safety and have regulatory implications.

In this work, we evaluated engineering Chinese Hamster Ovary (CHO) cell lines to create a new host cell line that would be resistant to MMV infection by modifying the major receptors used by the virus to enter cells. The goal was to engineer a host cell line resistant to MMV infection, while maintaining productivity and product quality profiles. Our strategy is outlined below.

**Figure 1:**
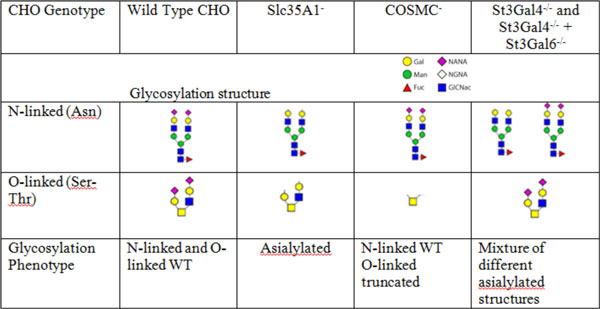
**ZFN mediated gene knock outs affecting terminal sialic acid on glycosylation structures tested for susceptibility to MMV infection**.

Attachment to a cell surface receptor is a key first step in the infection cycle for viruses. While the exact functional receptor for MMV binding to CHO cell surface is unknown, sialic acid on the cell surface has been implicated [1]. Moreover, MMV has been shown to preferentially bind to α-2,3 sialylated glycans with a type-2 Galβ1-4GlcNAc motif. Our approach was to systematically knock out genes affecting sialylation and then challenge each cell line for their ability to resist viral entry. Figure 1 lists the genes that were knocked out by ZFN mediated cell line engineering, and the corresponding typical glycosylation phenotypes expected on N-linked and O-linked glycans.

**Figure 2:**
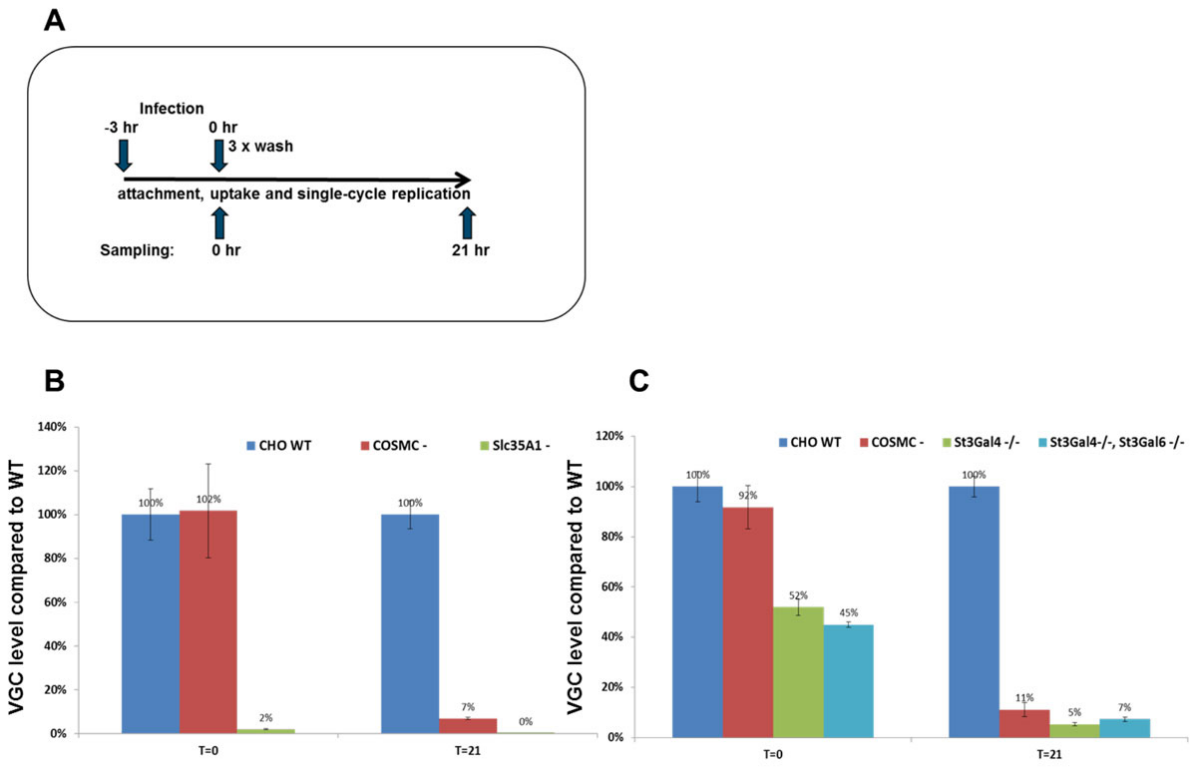
**(a) Schematic of the qPCR based assay for MMV virus attachment, internalization, and single cycle replication**. In brief the virus is incubated with the cells at an MOI of 0.1 for 3 hours. Samples are washed 3 times to remove excess unbound virus (T = 0) and harvested after an additional 21 hrs and analyzed by qPCR for presence of viral genome copies. (b) Viral genome copies compared to the WT at times T = 0 and T = 21 for the COSMC^- ^and the Slc35A1^- ^knock out clones, and (c) for the St3Gal4 ^-/- ^and the St3Gal4^-/- ^+ St3Gal6^-/- ^knock out clones.

MMV infectivity studies were conducted on the knock out clones identified in Figure 1 at various multiplicities of infection e.g. 0.1 viruses per cell, far higher than would be encountered in a fermenter infection. Infection of the cells was analyzed by PCR for the presence of viral genomic sequences as described in the schematic in Figure 2.

## Results

CHO cell lines engineered to express lower surface sialic acid demonstrate increased resistance to MMV infection compared to the wild-type controls. While the complete absence of sialic acid on the SLC35A1 knock-out cell line led to almost complete resistance to MMV infection, the COSMC knock out clones were about 10x more resistant to MMV infection. The knock out of the COSMC gene exclusively truncates O-glycosylation, leaving the N-glycosylation pathway and the terminal sialic acids intact. The lower levels of cell surface sialic acid however, were able to confer the 10x resistance to MMV infection. Moreover, the Slc35A1 knock-out appears to result in complete abrogation of binding of the virus to the cell surface, even at time T = 0. The COSMC knock-out on the other hand appears to affect virus internalization and replication rather than initial binding of the virus to the cell surface. In contrast, the St3Gal4 -/- and the St3Gal4-/- + St3Gal6-/- knock-out clones appear to affect both cell surface binding as well as the internalization of the MMV virus, with the cumulative effect being similar to that of the COSMC knock-out.

## Conclusions

Our data demonstrate that viral resistance against MMV virus can be incorporated into CHO production cell lines, adding another level of "defense", against the devastating financial consequences of this virus infection. While the importance of cell surface sialic acid for virus binding and internalization is clearly evident, further studies on the downstream steps of the MMV life cycle may provide further targets for interrupting and eliminating the infection process, thus conferring a higher degree of resistance.
